# *In silico *modelling of hormone response elements

**DOI:** 10.1186/1471-2105-7-S4-S27

**Published:** 2006-12-12

**Authors:** Maria Stepanova, Feng Lin, Valerie C-L Lin

**Affiliations:** 1 Bioinformatics Research Centre, Nanyang Technological University, 50 Nanyang Drive, Singapore 637553, Singapore; 2 School of Computer Engineering, Nanyang Technological University, 50 Nanyang Drive, Singapore 637553, Singapore; 3 School of Biological Sciences, Nanyang Technological University, 50 Nanyang Drive, Singapore 637553, Singapore

## Abstract

**Background:**

An important step in understanding the conditions that specify gene expression is the recognition of gene regulatory elements. Due to high diversity of different types of transcription factors and their DNA binding preferences, it is a challenging problem to establish an accurate model for recognition of functional regulatory elements in promoters of eukaryotic genes.

**Results:**

We present a method for precise prediction of a large group of transcription factor binding sites – steroid hormone response elements. We use a large training set of experimentally confirmed steroid hormone response elements, and adapt a sequence-based statistic method of position weight matrix, for identification of the binding sites in the query sequences. To estimate the accuracy level, a table of correspondence of sensitivity vs. specificity values is constructed from a number of independent tests. Furthermore, feed-forward neural network is used for cross-verification of the predicted response elements on genomic sequences.

**Conclusion:**

The proposed method demonstrates high accuracy level, and therefore can be used for prediction of hormone response elements *de novo*. Experimental results support our analysis by showing significant improvement of the proposed method over previous HRE recognition methods.

## Background

Steroid hormones are signal molecules that play essential roles in various physiological and pathological processes. In particular, one of the most important factors of regulation commonly applied in medical treatment is the use of hormones. Cancer treatment on early stages of tumor development is often associated with action of steroid hormones – progesterone [1] and estrogen [2]. Steroid hormones are believed to play an important role in the regulation of the development of breast cancer [3].

Hormone functions are mediated by hormone-specific receptors which are transcription factors [4]. The molecular effects of estrogen and progesterone are reflected by their receptor-regulated gene expression [5]. The overall mechanism of the gene expression regulation by steroid hormones in a cell does include several stages of reaction, and up to now, none of them is described in details. In the multi-stage regulation, the "primary target genes" may produce proteins involved in regulation of other genes, causing the second stage of "regulatory answer", and so on. Elucidation of the regulation network is further complicated by at least four possible hormone reactions [6]:

• Usual pathway: hormone receptors (HR) are activated by the correspondent hormones, and then bind directly to hormone response elements (HRE) within regulatory areas of DNA. Binding to regulatory elements induces changes in gene expression;

• Hormone receptors can be activated by different signaling molecules other than hormones (e.g. growth factors), and also interact with DNA of HREs;

• Activated hormone receptors bind indirectly to non-HRE sites via forming protein complexes together with other transcription factors; and

• Different signaling pathways through cellular membrane with effect on tissue responses (not related to gene expression).

We are especially interested in identification of the "primary target genes" of steroid hormone receptors and differentiation of them from the down-stream targets of these genes. The first step of investigation of the hormone-regulated gene expression network is the HRE prediction and analysis. Listed in the above, the first and second hormone reactions involve recognition of specific HREs.

In general, for the purpose of identification of transcription factor binding sites (TFBS), a number of tools have been reported, to name a few, MatInspector [7], Match [8] and MAPPER [9]. However, all of them, being appropriate for genome-scale analysis of trends and frequencies [10], provide too many false positives for investigation of singular sites to be reliable. We need to explore more accurate prediction methods with high sensitivity and specificity. First, a training HRE set from both literature and wet-lab experiments should be carefully constructed. Then a statistic model should be established with machine learning through the reported specific features of binding sites, in order to distinguish the HREs from other DNA sequences.

In this study, we exhaustively searched for training HREs from more than 200 literature sources. Two main criteria were taken into account: detection of DNA-receptor complex, usually with use of gel shift retardation assay [11]; and confirmed involvement into gene expression regulation, i.e. hormone treatment-associated changes either in mRNA or protein level, or in any plasmid construct activity.

We then examined the specific structure of binding sites of interest, which requires investigation of biological nature of hormone receptors. Some transcription factors, including the steroid hormone receptors, bind DNA as dimers [12]. Therefore, consensus hormone response element should include two constituent parts, or half-sites, which are usually separated by a short spacer.

**Figure 1:**
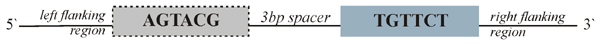
HRE consensus.

Two classes of steroid HREs have been characterized; Androgen, progesterone and glucocorticoid response elements (ARE/PRE/GRE), with a consensus sequence AGTACAnnnTGTTCT, has been studied the most extensively [13-15]. Mineralocorticoid receptors can also act via the HRE [16]. The estrogen response element (ERE) consensus sequence GGTCAnnnTGACC can be converted to a functional ARE/PRE/GRE by just changing two bases. The sequences are imperfect palindromes to which the receptor dimers bind [17]. Consensus sequence of the first class of HREs is shown in Fig. 1.

Finally, we developed a statistic model and implemented the tools for prediction of a specific group of steroid HREs including the above progesterone, androgen and glucocorticoid whose receptors were reported to share the same response elements [15]. Our approach involved extensive search for available experimental data and use of an adapted method of sequence representation, Position Weight Matrix, based on nucleotide position frequencies. Supervised machine learning was implemented to further improve the prediction accuracy.

## Results

### Experimentally verified HREs are used for training the statistic model

The data was collected from more than 200 literature sources and our in-house wet-lab experiments. Such a collection for HREs has no analogs in the current public and commercial databases of TFBS profiles. While a few of the regulatory elements are derived from genes of fish and birds, most of the sites are mammalian and 89% of all sites are from human or rodent genomic DNA.

It is also worth mentioning that most collections do not filter out confirmed binding sites from the recognized ones (i.e. when a DNA region is found to exercise promoter activity, regions similar to HRE consensus are predicted within the long promoter sequence by a computational method). Our aim is to collect sites with binding affinity, whatever their structure is. Therefore, only experimentally confirmed binding sites are included into our collection.

It is known that progesterone receptor, androgen receptor and glucocorticoid receptor tend to share the same binding sites on DNA (for a review, see [15]). While that was shown by many experiments, our statistic model further verified it and would serve as an additional control of appropriateness of the training HRE collection. None of the experimental methods allow detecting a response element to a single nucleotide precision. Therefore, after collection of the sequences, they were aligned [18] and HRE motifs within them were detected. Position weight matrices were computed with the aligned HREs for mono and di-nucleotide patterns. (For comparison, tri-nucleotide patterns were also examined.) Then, to check the above assumption of the same response element to be shared by three steroid hormone receptors, we used the chi-square criterion for homogeneity, and these three steroid hormones of interest do share the same response elements, with p-value of less than 10^-4^.

We joined the three position frequency matrices for AR, GR and PR into one Position Weight Matrix of Steroid Hormone Response Element, and used this matrix for further prediction of HREs. The joined matrix is given in Table 1.

### Combination of mono and di-nucleotide models significantly improves the accuracy

Based on the TFBS recognition strategy (details described in the section of Methods below), we implemented two modules: mono-nucleotide PWM1 and di-nucleotide PWM2. Each of them was characterized by non-lucrative trade-off between re-value and sensitivity.

With PWM1, a sensitivity of 86% was achieved only with re-value of 1 prediction per 1325 bp (threshold for calculated probability of 0.865), and re-value of 1:6 kb corresponds to sensitivity of 73% (threshold of 0.920). With PWM2, a sensitivity of 86% with re-value of 1:1025 bp (threshold of 0.725) vs. 71% with 1:5 kb (threshold 0.885) was achieved. None of them shows an impressive level of accuracy in recognition.

We then combined the results from two modules; that is, a motif is recognized if it is recognized by both modules. To our expectation, more false positives were removed. This can be achieved because while the first PWM measures only independent single nucleotide frequencies, the second also takes into account di-nucleotides which are often preserved in patterns other than simple combinations of independent nucleotides; for instance, CpG di-nucleotides occur in real genomic sequences much less than the expected rate of 1/4 × 1/4 = 1/16 in arbitrary nucleotides [19].

We indeed managed to eliminate a large number of the false positives, while holding the true positives at a reasonable level. Here sensitivity and re-values are functions of two variables, and it is possible to change them by moving in a two-dimensional space. In applications, to solve a specific problem, trade-off is made in regard with which direction to move in the space. In our case, we set the following values for recognition thresholds: PWM1 – 0.91, and for PWM2 – 0.79, in order to receive optimal combination of the sensitivity of 76% and random expectation of 1 hit per 7.14 kb.

### Supervised machine learning with neural network

We implemented an artificial neural network (ANN) to cross-verify the previous PWM-based predictions. ANN is to date the best tool to model individual prototypes. Due to its inherent nature, an ANN structure with enough connections and parameters to fit is able to mime almost any complex pattern.

During supervised learning and testing, most of sequences come to convergence to an exact YES/NO answer, but the rest requires setting up a threshold for decision making, when the relation of Euclidian distances from the actual ANN output to the YES (1;-1) and NO (-1;1) points is measured.

With the threshold value of 0.05, we achieved the specificity of 99.6%, and 8 of 661 HREs were misclassified (Details in the section of Discussion below). When the distance threshold was set to 0.005, sensitivity level decreased to 89%, i.e. 528 of 661 true HREs were indeed identified, but the specificity reaches as high as 99.8%.

We initially intended to use the ANN model only for cross-validation of the prediction, and through the machine learning process, we eventually further improved the prediction accuracy. The model now implies not only independent nucleotide positions but also a HRE sequence as whole. Thus, with an appropriate number of neurons, it is possible to reach very high sensitivity and specificity. Such an accurate model is approachable, provided that the exhaustive training procedure can be matched by computing power. We are currently in development of hardware acceleration of ANN training models [20,21].

## Discussion and Conclusion

One can hardly declare that each HRE predicted by the model is functional and involved in regulation of gene expression, though we have confirmed that our model can detect potential HREs with high confidence. Our correctly predicted HREs cover most of the microarray-verified progesterone primary target genes. The average number of the found PREs in promoter area for 380 human PR-responsive genes listed in [22] is 1.06; for the total set of human genes, this value is 0.62 HREs per promoter. Note that the promoter area is set from -3000 to +500 according to the annotated transcription start site. In the current work, Genbank build #35.1 has been used.

The highest probability of steroid hormone primary target gene was found for human MMP1 gene encoding for matrix metalloproteinase 1 (interstitial collagenase). Its promoter contains three predicted HREs, and two of them are adjacent and with a very high chance to be functional [23]. Steroid hormone progesterone was previously reported to reduce level of human MMP1 gene expression significantly [24]. The second significant PR-responsive gene NGRF was also reported to be progesterone-regulated [25].

The unsupervised learning of PWMs and the supervised learning procedure of ANNs imply different strategies for both modeling of HRE pattern and training the model. As for the structure, the probability score of PWM-predicted score is additive along the sequence of single model units (mono or di-nucleotides), while the ANN takes the input HRE sequence as a whole. That explains why, with comparable specificity value, ANN fits much close to a given training set of response elements. Major difference in learning lies in the strategy of binding site recognition. Neural network has both positive and negative patterns for the training, and thus the final recognition procedure during testing is a selection between two stable points – neutral or potential regulatory sequence. It can be understood why higher specificity value can be expected for the ANN model. However, as shown in our experiment, training a highly accurate ANN model takes a prohibitivly long time on our current workstation. Hardware acceleration such as Field-Programmable Gate Arrays may provide us with a solution.

Though, with use of ANN, we managed to model the HRE training set and separate it from the neutral DNA sequences quite well, some outliers were detected as well. They were found through non-consensus binding sites for progesterone, androgen and glucocorticoid receptors in the promoters and gene regions for a number of genes: rabbit uteroglobin gene [26], chicken lysozyme gene [27], porcine uteroferrin gene [28], pro-opiomelanocortin gene [29], murine c-myc gene [30], late leader of the control region of the human polyomavirus BK [31], gene promoter of two milk protein genes (β-casein and whey acidic protein) [32], human Na/K ATPase α 1 gene promoter [33], and mouse sex-limited protein enhancer [34]. The first three are progesterone-regulated genes, the next five are glucocorticoid primary targets, and the last one is associated with androgen activity. Unless they are experimental artifacts, the possible explanation could lie in the area of complex protein-DNA interaction which is beyond DNA sequence similarity itself, like secondary molecular structure of DNA or location of surrounding nucleosomes. Nevertheless, more sensitive procedures should be implemented. The subspace of HREs looks like to be non-uniform and can be clustered into different types [35], possibly avoiding more false positive in future model development.

In conclusion, our proposed model for steroid receptor binding sites prediction can be used for determination of androgen, progesterone and glucocorticoid primary target genes, detection of steroid hormone response elements *de novo*, and evaluation of known HREs. It is a crucial starting-point for reconstruction of the global hormone-regulated gene expression network, which is indeed a great challenge for both molecular biology and life science in general.

## Methods

### Unsupervised training for the HRE model

As an implementation of unsupervised learning algorithm for HRE modeling, an adapted position weight matrix approach is developed in this study.

#### • The Position Weight Matrix algorithm

We start with a statistic model of position weight matrix (PWM) which was first described by Quandt et al. [36]. We adapted the concept for recognition of HRE patterns. To justify if a given sequence is a PRE, we compare this sequence with a set of experimentally validated sequences. The similarity score of the comparison is proportional to the sum of all the results of position comparisons. For comparing a sequence to a matrix, we develop following processes:

A) Calculation of relative conservation for each position *i *in the matrix:



where *P(i, b) *is relative frequency of letter *b *in position *i*.

This relative conservation is proportional to the information content for each position, which, in turn, is indirectly concerned with nucleotide to amino acid binding energy [37]. As can be easily calculated, it takes value of 0 when nucleotide distribution on a particular position is uniform and demonstrates no preservation. The value of 1 is reached in the case of strong conservation of a particular nucleotide. The normalization factors were selected in order to vary C_i _strictly from 0 to 1. If for a particular position, an outcome of 'gap' is not rated, then in the above, coefficients 5 should be changed into 4; that is, it is always the number of possible outcomes for each position.

B) Calculation of the matrix similarity coefficient which represents resemblance of a given sequence and the pattern. The pattern is represented by the position frequency matrix.



where *b *is the *i*^*th *^letter of the sequence, and *score(i, b) *is the element of the position frequency matrix located in the row *i *and corresponding to the nucleotide *b*.

As can be seen, the higher MS coefficient is, the higher correspondent *score(i, b) *values will be; thus, higher MS values correspond to the sequences which consist of more frequent nucleotides. Finally, the higher MS value is, the closer the sequence is to the training set of experimentally validated binding sites.

The matrix similarity reaches 1 only if the candidate sequence corresponds to the most conserved nucleotide at each position of the matrix. Multiplying each score by the C_i _value emphasizes the fact that mismatches at less conserved positions are more easily tolerated than mismatches at highly conserved positions.

#### • Mono and di-nucleotide position weight matrices

In the modeling of PRE recognition for a given sequence two matrix similarity coefficients are calculated: MNMS (mono-nucleotide matrix similarity) and DNMS (di-nucleotide matrix similarity). Before calculating these coefficients, the sequence is aligned with consensus HRE. After all, for calculating the matrix similarity coefficients, only aligned sequence is used. However, it may contain one or more gaps after alignment procedure.

The first coefficient calculation is a simple comparison of mono-nucleotide position frequency matrix with the aligned sequence exactly as described above.

The second comparison requires prior preparation. A nucleotide sequence is to be pre-processed for appropriate comparison with di-nucleotide position frequency matrix. Alphabet of existing di-nucleotides consists of 25 elements (four different nucleotides and a gap in all possible combinations). Latin alphabet contains enough different letters to reconstruct one-to-one conformity, in which every di-nucleotide corresponds to a single letter of the new alphabet.

Then, for a sequence acquired as a result of this conversion, the matrix similarity coefficient is calculated exactly as in the above for mono-nucleotide frequency matrices, but the matrix in use now is the dinucleotide frequency one. The only correction is the change of normalization coefficients. Because the number of different di-nucleotides (and the corresponding number of letters in the newly implemented alphabet) is as many as 25, it is necessary to change 4 or 5 to 16 or 20 or 25, depending on whether a gap symbol is assumed in any position of the di-nucleotide.

Once these two coefficients have been calculated, the decision-making procedure is implemented. It uses cut-off levels for each of two coefficients. These cut-off levels must be predetermined by some tuning methods or cross-validation.

If for a given sequence, the MNMS is greater than the cut-off level for this value, and  DNMS exceeds its threshold as well, then it is a HRE.

For each recognized HRE the most similar element of training set is defined. It is also indicated if such a sequence of the right half-site (which is expected to be highly conserved) is presented in any of found experimentally validated HREs. This is important in the case of rather large and representative training set used, when absence of a given sequence in the sample might be a valuable indicator for tuning the model.

### Supervised machine learning

Feed-forward neural network is then used for cross-verification of the predicted HREs.

#### • Input/output representation

The collected dataset consists of a number of DNA sequences in 4-letter alphabet Ω = {A, C, G, T}. In the above position frequency based model, the letters are annotated as different, independent and equidistant states. However, the neural network model works with digital numbers. The space of numbers is one-dimensional, so if we confront all 4 nucleotides with numbers, they are not equidistant any more, and therefore we bring some artefacts to our model. After a few tests we found out that the artefacts of modelling using one-dimensional performance of input nodes are quite critical for the accuracy. Therefore, we implement the "one-hot" representation for DNA encoding.



For Yes/No decision, it is enough to represent the output as a single bit. However, for the purposes of distinguishing Androgen, Progesterone and Glucocorticoid response elements (which form our dataset of HREs), or any other clusters of HREs (as the HREs are definitely not a uniform subspace of DNA sequences), we present the output as a vector. In particular, the Yes/No output is a 2-vector: Yes = (1,-1) and No = (-1,1). In this case, the movement of output is in the two-dimensional space and allows more flexibility.

#### • The neural network structure

With the input as 4-dimensional vectors, for a 15 bp-long HRE, we have 60 input nodes. The neural network theory [38] suggests that for the confident learning the number of degrees of freedom, or weights to fit, be at most half of the number of constrains (the inputs). Hence, in the case of one hidden layer and a dataset of about 7000 positive and negative HREs, we should limit the number of hidden layer neurons to about 50. Thus, we have 60 × (50+1) weights of the hidden layer and (50+1) × 2 weights of output layer (plus one is for a bias term), total about 3000. In the case of two hidden layers, the maximum number of neurons on each layer is about 40. However, we found use of two layers is excessive for the current problem.

**Figure 2:**
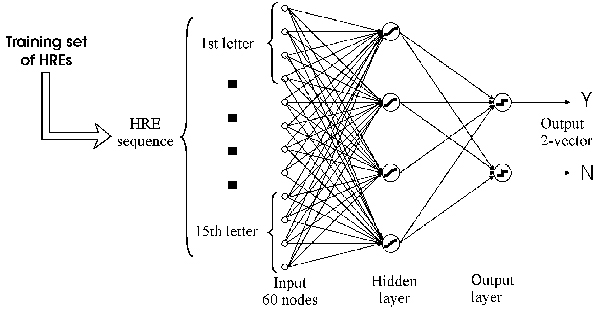
Artificial Neural Network for PRE recognition.

In our neural network model, bipolar sigmoid functions are used for implementation of all layers. The whole network structure is illustrated in Fig. 2.

#### • Back-propagation learning

For training of the model, the back-propagation learning is implemented: for each exemplar pattern from the training set, find difference of the weights for the output layer, back-propagate the difference to the hidden layer, then find the difference of the weights for the hidden layer, and finally modify all the weights of the network.

The equation of weights adjustment *for each neuron *is:

**w**^t+1 ^= **w**^t ^+ α × δ **x **    (4)

where **w**^t+1 ^is a vector of weights for a particular neuron at the t^th ^step of learning, α^t ^is the learning parameter at the t^th ^step (0 < α^t ^< 1 ∀ t > 0), the delta value *for each neuron *is calculated as follows:





where d^*t *^and *o*^*t *^represent the desired and currently obtained outputs of the neuron respectively, **x **is the input to the layer being considered (either hidden or output), *u*^*t *^= **w**^t^**x **is the synaptic input to the neuron, and *f*(*u*^*t*^) is the activation function of the neuron. Also, for the back-propagated delta value, K is a number of neurons on the output layer, *w*_*h*->*k *_is the weight coefficient of the connection between h^th ^neuron of hidden layer and k^th ^neuron of the output layer, δ_k _^output ^is a delta value for the k^th ^neuron of the output layer calculated as shown by formula (5).

The back-propagation is terminated when error tolerance for the accuracy of 99% is satisfied, the desired number of epochs is passed, or the error plateau is reached.

Learning rate parameter α regulates the stride of gradient descent algorithm for minimization of the learning error. The higher the learning rate is the faster convergence goes. But with a very high learning rate there is a chance to jump over the minimum of error, or receive oscillations instead of steady state. On the other hand, a very low learning rate provides less chance to find the global minimum, and instead, it uses the first randomly found local minimum. Our solution is to adjust the learning parameter: if the current pass error is less than the previous one, we are moving in a right direction and can move a bit faster, increasing α by 5%. If we've jumped over the minimum and received larger error, the system goes back with smaller steps, and α^t ^is decreased by 30%.

For the supervised learning, a set of experimentally verified HREs is used with desired answer YES (1;-1), and a tenfold set of neutral DNA sequences is associated with desired answer NO (-1;1). Both training sets are large, therefore, in order for the neural network to avoid severe oscillations, we mix them; otherwise, while looking through several hundreds of the positive (negative) training set, the network may adapt itself towards the positive (negative) answers, without attention to the negative (positive) ones.

### Accuracy estimation

For accuracy estimation purpose, ten-fold cross-validation is used, 90% of the total dataset being the training set and the rest 10 % being used for testing purposes. This separation is repeated 10 times (with non-overlapping testing sets), and the average error values are calculated.

## Authors' contributions

As a part of her PhD study, Maria conducted literature survey, carried out data mining and developed software for this project, under the supervision of Lin Feng and Valerie. Lin Feng and Valerie have collaboratively defined the research objectives and initiated the data preparation and modelling work. The manuscript is a result of numerous rounds of discussion, correction and refinement of the initial draft by the authors. All the authors have read and approved the final manuscript.

## Figures and Tables

**Table 1:** Joined HRE Position Weight Matrix.

position	Nucleotide frequency				C_i _value, %
	A	C	G	T	
1	0,19	0,28	0,24	0,30	1,08
2	0,26	0,20	0,23	0,31	1,09
3	0,17	0,24	0,36	0,24	2,60
4	0,39	0,08	0,34	0,19	9,80
5	0,16	0,08	0,60	0,16	20,86
6	0,28	0,21	0,12	0,39	5,84
7	0,58	0,09	0,13	0,20	18,63
8	0,13	0,60	0,15	0,12	20,16
9	0,52	0,15	0,15	0,19	11,93
10	0,33	0,16	0,23	0,29	2,40
11	0,25	0,25	0,30	0,19	0,85
12	0,42	0,23	0,22	0,13	6,01
13	0,13	0,03	0,02	0,82	55,29
14	0,00	0,00	0,99	0,00	97,45
15	0,01	0,01	0,00	0,97	89,76
16	0,11	0,14	0,05	0,70	33,33
17	0,01	0,94	0,00	0,04	80,66
18	0,05	0,17	0,01	0,76	47,44
19	0,29	0,21	0,23	0,27	0,54
20	0,36	0,11	0,25	0,28	5,16
21	0,34	0,21	0,18	0,26	2,05
21	0,34	0,21	0,18	0,26	2,05
